# Pellagra – eine klinisch-dermatohistopathologische Differenzialdiagnose zu „Acne excoriée des jeunes filles“

**DOI:** 10.1007/s00105-023-05157-y

**Published:** 2023-05-11

**Authors:** Anna-Maria Forster, Stefan Zechmann, Tobias Plaza

**Affiliations:** 1 Poliklinik für Dermatologie und Venerologie, Universitätsspital Basel, Medizinische Universität Basel, Burgfelderstrasse 101, 4055 Basel, Schweiz; 2 Universitätsklinik für Innere Medizin, Universitätsspital Zürich, Zürich, Schweiz; 3 Plaza Kliniken, Oberlandstr. 100, 8610 Uster, Schweiz

## Anamnese

Eine 41-jährige Patientin wurde erstmalig mit einer seit über 20 Jahren bestehenden Akne in unserer Praxis vorstellig. Im Rahmen zahlreicher vorangegangener, dermatologischer Konsultationen führten topische Therapien und systemische Medikationen mittels 13-cis-Retinsäure, Clindamycin, Doxycyclin und Rifampicin nur bedingt und zu jeweils kurzfristiger Besserung des Hautzustandes. Unterdessen sei es aufgrund zunehmender gastrointestinaler und psychischer Beschwerden zu einer immer stärkeren Einschränkung der Lebensqualität gekommen. Ein reger Stuhlgang mit 2 bis 4 Entleerungen täglich sowie depressive Verstimmungen, Angst- und Schlafstörungen gipfelten nach internistischer Abklärung im sozialen Rückzug und einer engmaschigen, psychologischen Betreuung. Fixmedikationen, Nebenerkrankungen, Diäten, Alkohol- und Nikotinabusus oder Allergien wurden verneint. Familienanamnestisch litt die Mutter an einer hartnäckigen Form von Akne, die bis ins hohe Alter andauerte.

## Untersuchung

**Figure 1:**
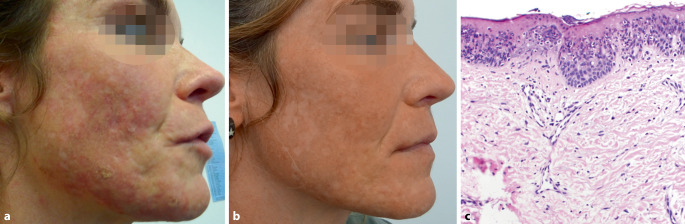

Es präsentierte sich uns eine agitierte, aber körperlich gesunde Frau in gutem Allgemein- und Ernährungszustand. Das Gesicht zeigte ein nahezu poikilodermes Hautbild mit multiplen schmerzhaften, teils exkoriierten Papeln auf flächig erythematösem Grund. Dazu imponierte eine atrophe Narbenbildung mit einer ausgeprägten postinflammatorischen Hyperpigmentierung, insbesondere an UV-exponierten Arealen ohne Hinweis für verstärkte Seborrhö oder Komedonen in der T‑Zone (Abb. 1).

## Diagnostik

Zum Ausschluss autoinflammatorischer oder photoaggravierter Dermatosen insbesondere bei scharf umschriebenen Hyperpigmentierungen und fehlenden Komedonen führten wir eine läsionale Stanzbiopsie für die Routinehistologie durch. Histologisch zeigte sich eine Atrophie der Epidermis mit fokaler Hyper‑, Parakeratose und Ballonierung der Keratinozyten nebst verstärkter Vaskularisierung und geringer entzündlicher Umgebungsreaktion ohne Zeichen einer Interface-Dermatitis (Abb. 1). Neben einer „Acne excoriée des jeunes filles“ musste aufgrund epidermaler Nekrosen und chronischer perivaskulärer Entzündungsreaktion an nahrungsbedingte Mangelerscheinungen wie Vitamin‑B_3_- oder Zinkmangel gedacht werden. Eine anschließende laborchemische Untersuchung bestätigte mit 7,7 µl/l einen Vitamin‑B_3_-Mangel während Eisen, Ferritin, Vitamin B_12_, Vitamin B_6_ und Zink im Normbereich lagen.

## Therapie und Verlauf

In Zusammenschau mit den klinisch-pathologischen und laborchemischen Befunden leiteten wir eine orale Vitamin-B-Komplex-Therapie mittels Becozym® forte-Dragées von Bayer (Schweiz) AG mit insgesamt 150 mg Nicotinamid/Tag ein. Nach Angaben der Patientin sei es bereits innerhalb weniger Tage zu einer Besserung der Schlaflosigkeit und Konzentrationsfähigkeit gekommen. Vier Wochen nach Einleitung der Therapie zeigten sich nicht nur die entzündlichen Hautläsionen abgeheilt, sondern die Diarrhö und der psychische Zustand stabilisiert (Abb. 1). Unter Berücksichtigung des typischen Symptomkomplexes einer Pellagra mit gastrointestinaler und neurologischer Symptomatik neben einem dermatitischen Hautbild wurde die Patientin unter laufender Substitutionstherapie zur weiteren Abklärung an das Institut für Gastroenterologie des Universitätsspitals Zürich überwiesen. Ob der langjährige Vitamin‑B_3_-Mangel im Falle unserer Patientin einer erworbenen intestinalen Resorptionsstörung oder einer bisher unbekannten genetischen Form eines „Morbus Hartnup“ zuzuordnen ist, konnte bisher nicht vollständig geklärt werden. Neben einem konsequenten UV-Schutz und einer Vitamin-B-Komplex-Erhaltungstherapie werden regelmäßige, klinische Kontrollen im Abstand von 3 Monaten in unserer Praxis fortgeführt. Eine Narbenkorrektur mittels fraktionierten CO_2_(Kohlendioxid)-Lasers kann auf dringenden Wunsch der Patientin erst nach mehrmonatiger Stabilisierung des Hautzustandes erwogen werden.

## Diskussion

Nicotinamid ist die Pyridin-3-Carbonsäureamid-Form von Niacin oder Vitamin B_3_ [1]. Niacin als Vorläufer der beiden Koenzyme Nicotinamid-Adenin-Dinukleotid (NAD) und NAD-Phosphat ist für zahlreiche oxidative Reaktionen unerlässlich [2]. Obwohl Nicotinamid aus der Aminosäure L‑Tryptophan über den hepatischen Kynurenin-Signalweg synthetisiert wird, kann der tägliche Bedarf dadurch nicht ausreichend gedeckt werden. Infolgedessen wird Niacin zu den essenziellen Vitaminen gezählt [3]. Die Metaboliten des Kynurenin-Signalwegs spielen in der immunologischen Abwehr und der Regulierung der Darmmotilität bis hin zur Funktion des zentralen Nervensystems (ZNS) eine wesentliche Rolle und können als eigenständige Neurotransmitter des ZNS angesehen werden, die Stimmung und das Angstverhalten beeinflussen [4]. Ein Mangel an Vitamin B_3_ verursacht die Systemerkrankung Pellagra [5], die durch die 3 Ds – „dermatitis“, „dementia“ „diarrhoe“ – und möglicherweise durch ein viertes „death“ gekennzeichnet ist [6]. Die klassischen Hautläsionen der Pellagra wurden erstmals 1771 von Frapolli beschrieben und werden aus dem Italienischen mit „raue Haut“ übersetzt [7]. Diese präsentieren sich typischerweise mit einer lokalisierten, erythematösen Dermatitis insbesondere an sonnenexponierten Arealen wie Gesicht, Hals, oberer Brustkorb (Casal-Halsband), Hand- und Fußrücken, die von Pruritus und einem „Hautbrennen“ begleitet wird. Nach einigen Wochen folgen eine Sebostase und Hyperpigmentierung mit anschließender Exfoliation. Der Mechanismus der Photosensibilität ist bisher ungeklärt, jedoch wird eine phototoxische Genese vermutet [8]. Extrakutane Manifestationen sind weniger spezifisch, aber in Assoziation mit Pellagra-typischen Hautveränderungen ausreichend, um eine Diagnose zu stellen. Die neurologische Symptomatik umfasst klassisch das Demenzsyndrom einer „Pellagra-Enzephalopathie“ kann sich jedoch ebenso in Form von Schlaflosigkeit, Delirium, Depression, Kleinhirn- oder extrapyramidalen Symptomen präsentieren [2]. Gastrointestinale Symptome reichen von unspezifischen Beschwerden wie Glossitis, Dysphagie, Nausea, Emesis oder Bauchschmerzen bis hin zu hartnäckiger Diarrhö, die in fortgeschrittenen Krankheitsstadien rasch zum Tod führen kann. In der heutigen Zeit ist das klinische Vollbild selten geworden, und nur einzelne dieser Manifestationen können vorliegen [6]. Ätiopathogenetisch werden bei Pellagra 3 verschiedene Mechanismen unterschieden:

1. eine Änderung der Tryptophanaufnahme, -resorption und/oder -verstoffwechselung im Rahmen von persistierender Diarrhö, Anorexie, chronischem Alkoholabusus, Malabsorption, Leberzirrhose, Morbus Hartnup oder einer HIV(humanes Immundefizienzvirus)-Infektion;
2. eine Änderung im Serotoninstoffwechsel durch hormonaktive Karzinoiderkrankungen;
3. iatrogen/pharmakologisch durch die Einnahme von Medikamenten wie Isoniazid, Ethionamid, 6‑Marcaptopurin, Acetylpyridin, Thiosemicarbazon, Methopterin, 5‑Fluorouacil, Azathioprin, Pyrazinamid, Hydantoin, Phenobarbital oder Chloramphenicol [8].

Die Behandlung der Pellagra basiert zunächst auf dem Verzicht prädisponierender Faktoren sowie einer Substitutionstherapie mittels Niacin mit zumindest 300 mg/Tag. Darüber hinaus sollte präventiv auf eine ausgewogene Ernährung mit Niacin-reichen Lebensmitteln wie Kleie, Eier, Fleisch, Geflügel, Fisch, Hülsenfrüchte und Samen geachtet werden, nur um einige der Quellen zu nennen [8]. Ein Konsens bezüglich Dosierung, Form und Dauer der Behandlung ist nicht bekannt. Unter der Substitutionstherapie verschwinden Hautläsionen, gastrointestinale und neurologische Beschwerden im Allgemeinen innerhalb weniger Stunden bis Tage wie im Fall unserer Patientin. Die Diagnose einer Pellagra kann somit mit einem raschen Therapieansprechen retrospektiv suffizient bewiesen werden [9].

## Fazit für die Praxis

- Der vorliegende Fall verdeutlicht, dass bei einem Symptomkomplex aus Dermatitis, psychisch/neurologischen und gastrointestinalen Beschwerden eine Pellagra in Betracht gezogen werden muss.
- Dermatohistopathologisch kommen neben einer „Acne excoriée des jeunes filles“ differenzialdiagnostisch Hypovitaminosen wie Vitamin‑B_3_- und Zinkmangel infrage.
- Eine gründliche und umfassende Anamnese insbesondere bei hartnäckigen und therapieresistenten Fällen von „Acne excoriée des jeunes filles“ ist obligat.
